# Antimicrobial potential of endocannabinoid and endocannabinoid-like compounds against methicillin-resistant *Staphylococcus aureus*

**DOI:** 10.1038/s41598-018-35793-7

**Published:** 2018-12-06

**Authors:** Mark Feldman, Reem Smoum, Raphael Mechoulam, Doron Steinberg

**Affiliations:** 1 Biofilm Research Laboratory, Institute of Dental Sciences, Faculty of Dental Medicine, The Hebrew University of Jerusalem, Jerusalem, Israel; 2 The Institute for Drug Research, School of Pharmacy, The Hebrew University of Jerusalem, Jerusalem, Israel

## Abstract

Infections caused by antibiotic-resistant strains of *Staphylococcus aureus* have reached epidemic proportions globally. Staphylococcal biofilms are associated with increased antimicrobial resistance and are generally less affected by host immune factors. Therefore, there is an urgent need for novel agents that not only aim at multidrug-resistant pathogens, but also ones that will act as anti biofilms. In the present study, we investigated the antimicrobial activity of the endocannabinoid (EC) anandamide (AEA) and the endocannabinoid-like (EC-like), arachidonoyl serine (AraS) against methicillin resistant *S. aureus* strains (MRSA). We observed a strong inhibition of biofilm formation of all tested MRSA strains as well as a notable reduction of metabolic activity of pre-formed MRSA biofilms by both agents. Moreover, staphylococcal biofilm-associated virulence determinants such as hydrophobicity, cell aggregation and spreading ability were altered by AEA and AraS. In addition, the agents were able to modify bacterial membrane potential. Importantly, both compounds prevent biofilm formation by altering the surface of the cell without killing the bacteria. Therefore, we propose that EC and EC-like compounds may act as a natural line of defence against MRSA or other antibiotic resistant bacteria. Due to their anti biofilm action these agents could also be a promising alternative to antibiotic therapeutics against biofilm-associated MRSA infections.

## Introduction

Infectious diseases have been associated with morbidity and mortality throughout the history of mankind. Antibiotics were considered the ultimate weapon against bacteria. However, over time, bacteria have developed mechanisms to overcome the killing effect of antibiotics. Moreover, the bacterial pathogens’ ability to adapt to and overcome the challenges of antibiotics has been dramatically enhanced of late. Not only are rates of bacterial resistance to individual drugs or drug classes a concern, but the prevalence of multidrug-resistant strains (resistant to three or more drug classes) is an even more serious therapeutic challenge^1^.

Some of the more problematic drug-resistant pathogens encountered today include methicillin-resistant *Staphylococcus aureus* (MRSA), multidrug-resistant *Streptococcus pneumoniae*, and vancomycin-resistant *Enterococcus* spp. among the gram-positive bacteria, and multidrug-resistant *Acinetobacter baumannii*, *Klebsiella pneumoniae*, *Escherichia coli*, and *Pseudomonas aeruginosa* among the gram-negative bacteria^2^.

A multidrug-resistant phenotype can arise in bacteria through the acquisition of multiple acquired resistance mechanisms due to environmental pressure. These resistance factors can stem from mobile genetic elements, a combination of acquired and chromosomally encoded resistance mechanisms, or accumulation of multiple chromosomal changes over time. Another means for bacteria to evolve resistance to antibiotics is a single or poly-mutational event leading to overexpression of a multidrug-resistance mechanism, i.e., an efflux pump, or genes encoding a specific drug-deactivating enzyme^3^.

*S. aureus* are not naturally pathogenic and commonly colonize human epithelia. However, infections can occur on epithelial surfaces, ranging from pimples and impetigo to pneumonia and meningitis^4^. Furthermore, pathogenicity can develop through infection of *S. aureus* in the bloodstream, and these infections are of great medical importance due to their prevalence and virulence^5,6^.

Infections caused by antibiotic-resistant strains of *S. aureus* have spread globally and reached epidemic proportions worldwide^7^. In addition to the increasing prevalence and incidence of community-associated methicillin-resistant *S. aureus* (CA-MRSA), the strains appear to be especially virulent^8^. Overwhelming and tissue-destructive infections, such as necrotizing fasciitis and fulminant, necrotizing pneumonia, have been associated with CA-MRSA strains^9^. Moreover, MRSA can colonize the health care units of hospitals and clinics^10–12^ and therefore are of specific public danger.

All implanted medical devices are susceptible to colonization by staphylococci and staphylococcal biofilm-associated infections, from indwelling catheters to prosthetic heart valves, cardiac pacemakers, contact lenses, cerebrospinal fluid shunts, joint replacements and intravascular lines^13^. Damaged host tissue is also a risk factor for developing biofilm-associated infection^14^.

Biofilms are highly structured surface-associated communities of microorganisms that are enclosed in a self-produced protective extracellular matrix^15–17^. Typically, these biofilms are associated with increased resistance to antimicrobial compounds^17^ and are generally less affected by host immune factors. Bacterial biofilms are known to cause more than 75% of microbial infections in humans^18^. Therefore, there is an urgent need for antibacterial agents that not only target multidrug-resistant pathogens, thereby decreasing the use of antibiotics and hence their side effects, but also eliminate biofilms. An important potential strategy to help combat the resistance problem involves the discovery and development of new active agents capable of partly or completely suppressing bacterial resistance mechanisms^19^.

The endocannabinoid system (ECS) is composed of endocannabinoids (ECs) and enzymes for their synthesis and degradation, as well as the cannabinoid receptors CB1 and CB2, which are widely distributed throughout the body. Cannabinoid receptors are activated by different ligands that are either endogenous, such as the ECs, or exogenous, such as delta-9-tetrahydrocannabinol (THC) present in *Cannabis sativa* and synthetic cannabinoid-like compounds^20,21^. The ECS has been shown to affect numerous physiological processes, including appetite, the immune response, sleep, bone density, and neuroprotection. The ECS is thought to be a neuromodulator^22,23^ and an immunomodulator^24^. Functionally, the activation of cannabinoid receptors has been shown to play a role in the activation of GTPases in macrophages and neutrophils. These receptors have also been implicated in the proper migration of B cells into the marginal zone and the regulation of healthy IgM levels^25^. The EC anandamide (AEA) and EC-like arachidonoyl serine (AraS) are endogenous constituents in mammals and some other animal species^26,27^. AEA binds to both cannabinoid receptors; AraS does not bind to the receptors, but its neuroprotective activity can be blocked by CB2 receptor antagonists, indicating that it is part of the ECS^28^.

There is limited information concerning the role of the ECS during infection, particularly against invading bacteria. A previous study showed antimicrobial effects of *C. sativa* extracts on different pathogens^29^. Another work demonstrated strong antibacterial activity of selected cannabinoids against MRSA strains, indicating the therapeutic potential of some cannabinoids for the treatment of antibiotic-resistant *S. aureus*^30^. We have previously shown that the potent synthetic cannabinoid receptor agonist HU-210 reduces biofilm formation in a strain of *Vibrio harveyi*^31^. As biofilm formation is one of the routes of bacterial resistance to antibiotics, we posited that EC and EC-like compounds may also show antibacterial/antibiofilm activity and may represent one of the body’s reactions to invasion of bacteria and to bacteria which are resistant to antibiotics.

## Results

### Effect of AEA and AraS on bacterial growth

Using the standard broth microdilution methods, we evaluated the MICs of the tested compounds. The EC AEA did not exhibit inhibitory effect on any of the tested MRSA strains’ growth up to a concentration of 256 µg/ml (Table 1). In contrast, MICs of EC-like AraS varied with different strains. MICs of >256 µg/ml, 32 µg/ml and 128 µg/ml were detected against MRSAs 33592, CI and 43300, respectively (Table 1). The MIC of the antibiotic gentamycin, as a control, was 256 µg/ml for the CI and 43300 strains, and 128 µg/ml for the 33592 strain.

**Table 1:** MICs of the agents (µg/ml) toward MRSA strains.

	MRSA 33592	MRSA CI	MRSA 43300
AEA	>256	>256	>256
AraS	32	>256	128

### Effect of AEA and AraS on biofilm formation

**Figure 1:**
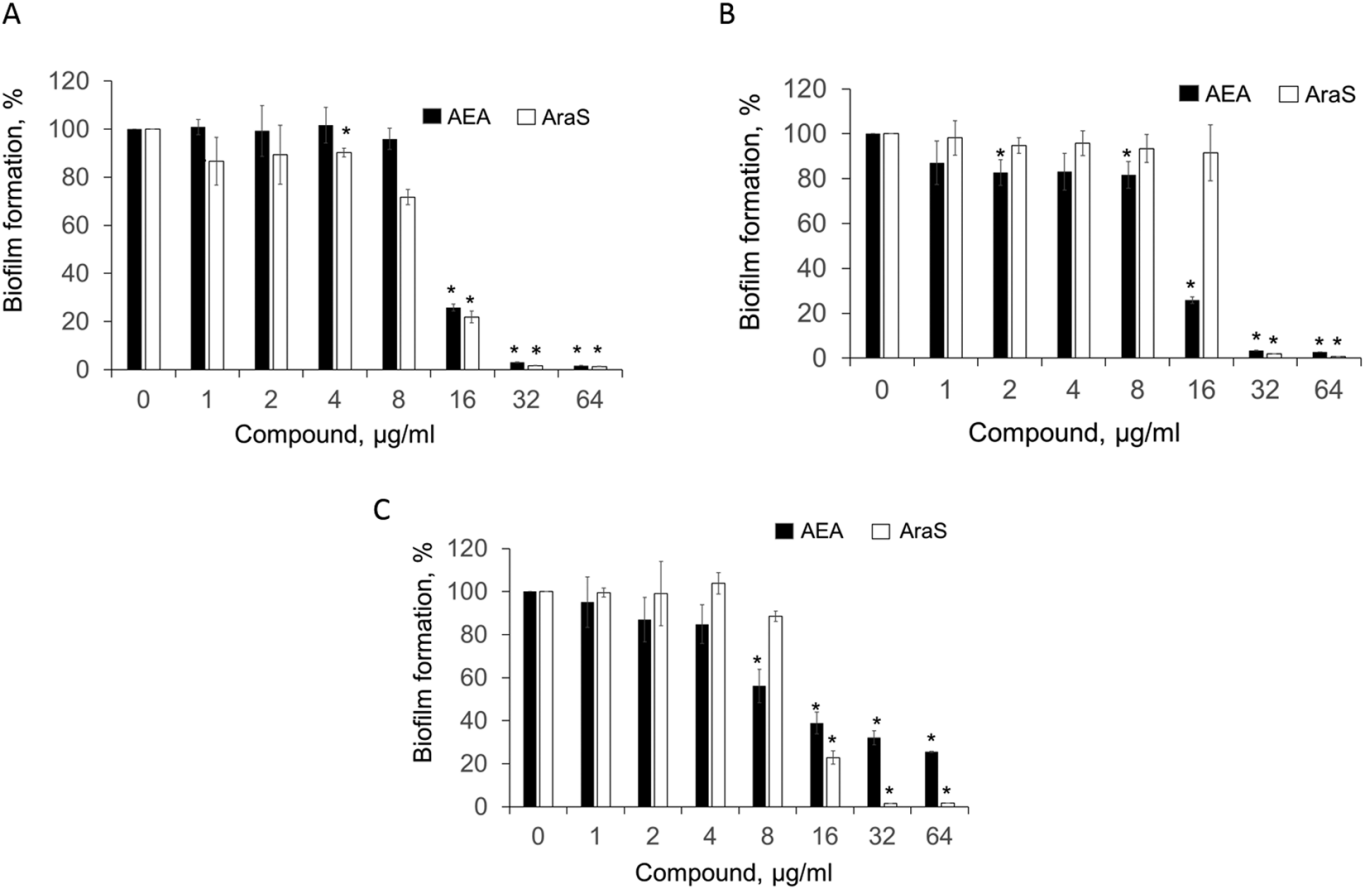
Effect of the agents on biofilm formation. Biofilms of MRSA 33592 (**A**); MRSA CI (**B**); MRSA 43300 (**C**) were grown with the presence of AEA or AraS at concentrations of 0–64 µg/ml. Quantification of the biofilm formation was conducted by crystal violet staining. Minimal Biofilm Inhibitory Concentration (MBIC) was determined as the lowest concentration of the tested compounds showing biofilm inhibition by 90% as compared to untreated control. *Significantly lower than the value for the untreated control (*P* < *0.05*).

Both, AEA and AraS exhibited pronounced dose-dependent inhibitory effects on biofilm formation of all tested MRSA strains (Fig. 1). Total biofilm inhibition of MRSA strains CI and 33592 was detected at a dose of 32 µg/ml AEA, which is more than 8-fold lower than its MIC against these strains (Fig. 1A,B). The biofilm formation of strain MRSA 43300 was also affected by AEA, but to a lesser extent. Although no MBIC was detected, AEA at doses of 8 µg/ml, 16 µg/ml, 32 µg/ml and 64 µg/ml was able to reduce the biomass of these bacteria in a dose-dependent manner by 44%, 64%, 68%, and 75%, respectively, as compared to the untreated control (Fig. 1C). AraS demonstrated MBIC = 32 µg/ml for all strains (Fig. 1A–C), while at the lower concentration of 16 µg/ml it was already capable of dramatically decreasing the biomass of MRSA 33592 and 43300 by almost 80% as compared to the untreated control (Fig. 1A–C). The MBIC of the antibiotic gentamycin, as a control, was 256 µg/ml for the CI and 43300 strains, and 128 µg/ml for the 33592 strain.

### Effect of AEA and AraS on pre-formed biofilm

In addition to the inhibitory effect of the tested compounds on bacterial biofilm formation, both ECs demonstrated strong attenuation of mature biofilm (Table 2). Metabolic activity of pre-formed biofilms of all tested MRSA strains was reduced dose-dependently by AEA and AraS at sub-MICs. Metabolic activity of staphylococcal mature biofilms was reduced by 20%–23% by 16 µg/ml AEA (Table 2), while an increased dose of AEA (64 µg/ml) caused more than 50% reduction of metabolic activity as compared to untreated biofilms (Table 2). AraS also exhibited high potency for pre-formed biofilm alteration of two of the tested MRSA strains, CI and 43300 compared to their untreated controls (Table 2). MRSA 33592 was more tolerant to AraS compared to its untreated control (Table 2). A concentration of 4-fold less than the minimal tested dose did not exhibit an effect on the pre-formed biofilm of all tested bacterial strains (data not shown).

**Table 2:** Biofilm eradication (in %) by the agents as compared to untreated controls (100%).

MRSA strain	Compound			
CI	AraS, 16 µg/ml	AraS, 64 µg/ml	AEA, 16 µg/ml	AEA, 64 µg/ml
	29 ± 4.1*	61 ± 2.3*	23 ± 0.1*	51 ± 2.7*
33592	AraS, 4 µg/ml	AraS, 16 µg/ml	AEA, 16 µg/ml	AEA, 64 µg/ml
	21 ± 1.4	33 ± 2.1*	20 ± 7.3	64 ± 2.2*
43300	AraS, 8 µg/ml	AraS, 32 µg/ml	AEA, 16 µg/ml	AEA, 64 µg/ml
	9 ± 1.0	52 ± 0.1*	20 ± 2.2*	58 ± 4.2*

*Significantly lower than the untreated control (*P* < *0.05*).

### Effect of AEA and AraS on cell-surface hydrophobicity

Both, AEA and AraS at sub-MICs altered the cell-surface characteristics of all tested MRSA strains. AEA at 16 µg/ml dramatically reduced the HI of CI, 33592 and 43300 strains by 4-fold, 3-fold and almost 5-fold, respectively (Table 3). AraS was less potent, but at sub-MICs still showed decreases in HI of MRSA 33592 and 43300 by 3-fold, and MRSA CI by less than 3-fold (Table 3).

**Table 3:** Effect of the agents on cell-surface hydrophobicity. The hydrophobicity index (HI) of MRSA cells incubated for 20 min with AEA or AraS.

	MRSA alone	compound treatment	
	CI	AEA, 16 µg/ml	AraS, 16 µg/ml
HI,%	87 ± 3	22 ± 0.6	38 ± 2
	33592	AEA, 16 µg/ml	AraS, 4 µg/ml
HI,%	92 ± 3	31 ± 2.5	29 ± 1.6
	43300	AEA, 16 µg/ml	AraS, 32 µg/ml
HI,%	90 ± 2.7	19 ± 0.74	29 ± 3.6

### Effect of AEA and AraS on spreading ability of MRSA

**Figure 2:**
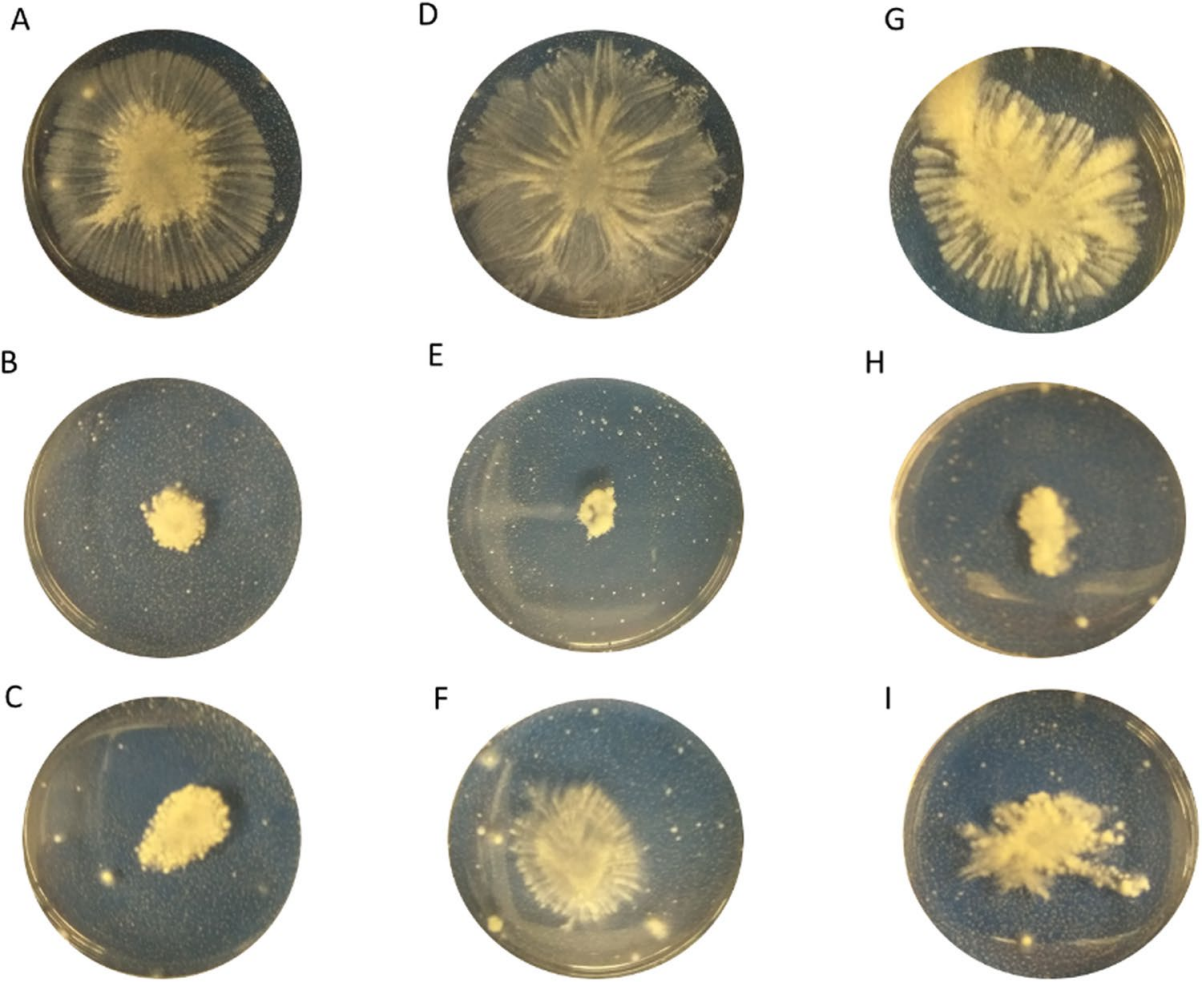
Spreading ability of MRSA in the presence of the agents. The assay was performed in a petri dish, on TSB containing 0.3% (w/v) agar-agar powder and sub-MIC of the tested compounds. The overnight culture of MRSA strains was point inoculated at the center of the agar medium and incubated for 24 h at 37 °C. Panels A, D, G-MRSA CI, MRSA 33592, MRSA 43300 untreated controls; Panels B, E, H- MRSA CI, MRSA 33592, MRSA 43300 treated with AEA at 64 µg/ml; Panels C, F, I- MRSA CI, MRSA 33592, MRSA 43300 treated with AraS at 64 µg/ml, 16 µg/ml, 32 µg/ml.

**Figure 3:**
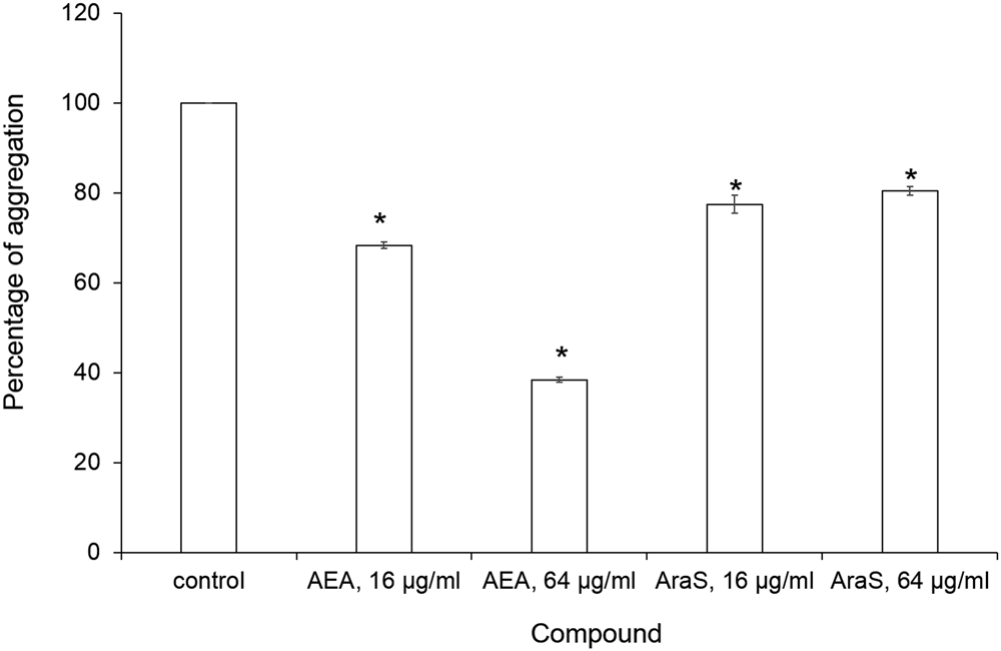
Cell aggregation of MRSA CI strain in the presence of the agents. MRSA cells (CI strain) were inoculated with the tested agents for 24 h. After centrifugation, cells were resuspended in PBS in clean glass tubes and allowed to stand for 24 h at room temperature. Next, supernatants were gently aspirated and aggregated pellets were resuspended in PBS. Turbidity of aggregates was measured at OD_595_ using spectrophotometer. The relative aggregation of the treated samples was presented as a percentage compared to untreated control (100%). *Significantly lower than the value for the untreated control (*P* < *0.05*).

All tested MRSA strains demonstrated strong ability to spread on the agar (Fig. 2A,D,G). Both agents, AEA and, with less impact, AraS—were able to inhibit colony spread. AEA at 64 µg/ml reduced the colony diameters of CI, 33592 and 43000 strains by 88% (Fig. 2B, Table 4), 84% (Fig. 2E, Table 4), and 73% (Fig. 3H, Table 4), respectively, as compared to untreated controls (Fig. 2A,D,G). AraS at sub-MICs was able to inhibit colony spread of CI, 33592 and 43000 strains by 64% (Fig. 2C, Table 4), 65% (Fig. 2F, Table 4), and 46% (Fig. 2I, Table 4), respectively, as compared to untreated controls (Fig. 2A,D,G).

**Table 4:** Percent inhibition of spreading ability. Levels of the spreading ability were determined by measuring diameters of the spreading and then compared to untreated control.

MRSA strain	Compound	
CI	AEA, 64 µg/ml	AraS, 64 µg/ml
	88 ± 1.9*	64 ± 2.5*
33592	AEA, 64 µg/ml	AraS, 16 µg/ml
	84 ± 1.8*	65 ± 3.4*
43300	AEA, 64 µg/ml	AraS, 32 µg/ml
	73 ± 2.6*	46 ± 2.8*

*Significantly lower than the untreated control (*P* < *0.05*).

### Effect of AEA and AraS on cell aggregation

AEA dose-dependently reduced the formation of bacterial aggregates at 16 and 64 µg/ml by 32% and 62%, respectively, as compared to untreated control (Fig. 3). AraS also demonstrated significant inhibitory activity on MRSA aggregation, but to a lesser extent. It decreased bacterial aggregation at 16 and 64 µg/ml by approximately 20%, as compared to untreated control (Fig. 3).

### Effect of AEA and AraS on membrane potential (MP)

**Figure 4:**
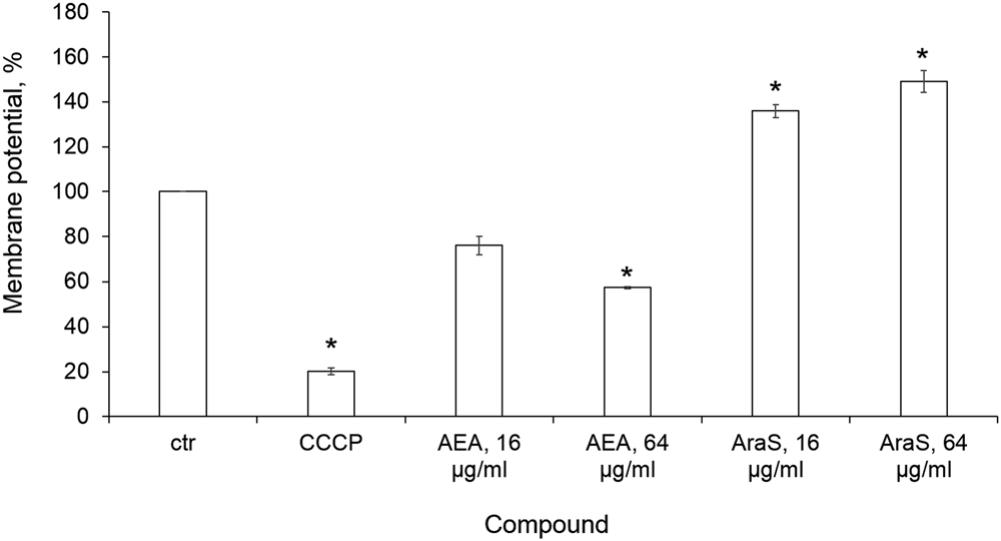
The effect of the agents on MP. Overnight grown MRSA CI cells were incubated with DiOC_2_(3). From this mixture, 10 μl were added to 40 μl reaction containing agents at sub-MIC or PBS (untreated control) and read immediately in fluorimeter. Excitation was read at 450 nm and emission at 690 nm. The difference in fluorescence between tested compounds treated and untreated control cells was detected at 690 nm and calculated as a percentage compared to untreated control (100%). *Significantly lower than the value for the untreated control (*P* < *0.05*).

Both agents at sub-MICs affected the MP of MRSA CI in a dose-dependent manner, but their effects differed. AEA at 16 µg/ml and 64 µg/ml decreased staphylococcal MP by 24% and 43%, respectively, as compared to the untreated control, thus causing bacterial membrane depolarization. As expected, MP was dramatically lowered by the known proton ionophore, CCCP, by 80% as compared to the untreated control (Fig. 4). In contrast to the depolarizing activity of AEA, bacterial exposure to AraS led to hyperpolarization of the MRSA membrane. This agent increased MP at 16 µg/ml and 64 µg/ml by 36% and 49%, respectively, as compared to the untreated control (Fig. 4).

## Discussion

Bacterial infections, particularly those related to biofilm-associated antibiotic resistance, are a serious clinical problem world-wide. EC are endogenous compounds known to affect physiological pathways in the body. In the current study, both the EC, AEA and the EC-like compound AraS were found to exhibit also anti microbial effect as anti-MRSA biofilm activity. However, AEA but not AraS had no inhibitory effect on any of the tested MRSA strains. Further, both compounds were able to impair already established staphylococcal biofilm at sub-MICs. Mature biofilms are generally difficult to disrupt and established MRSA biofilms are much more resistant to antimicrobial agents than planktonic MRSA^32,33^.

Colony spreading ability, an essential virulence determinant of *S. aureus*, was strongly affected by both agents at sub-MICs. AEA notably reduced the spread of all tested MRSA isolates by 73%–88%, whereas AraS was less effective, decreasing spread by 46%–65%. Motility mechanisms have previously been shown to play an important role in bacterial virulence and colonization^34–36^. Indeed, colony spreading ability of *S. aureus* on soft agar increases with increasing biofilm-forming activity^37^, suggesting that the spreading ability of *S. aureus* is an important factor for host and prosthetic material colonization. Interestingly, both tested compounds were able to inhibit colony spread without impairing bacterial viability.

In the absence of host factors, staphylococci can form multicellular clusters, known as aggregation. Bacterial cell aggregation is one of prerequisites for biofilm development^38^. In *Staphylococcus* spp., this process is associated with the production of extracellular polysaccharide intercellular adhesin, a compound that is important for intercellular adhesion; this is a necessary step for the accumulation phase of biofilm formation following initial attachment to a surface^39–41^. Moreover, protein A, which is responsible for the aggregative *S. aureus* phenotype, has been shown to induce biofilm formation under static and flow conditions^42^. We showed that the EC AEA dose-dependently inhibits aggregation of the MRSA CI strain, whereas EC-like AraS had no effect.

Microbial cell surface properties such as hydrophobicity play a crucial role in bacterium–host cell interactions, as well as in bacterial adhesion as an initial and critical step in biofilm development^43–45^. Various agents inhibit biofilm formation by interfering with bacterial cell-surface hydrophobicity^46–50^. In the present study, both ECs at sub-MICs were able to significantly modify the cell-surface properties of all tested MRSA strains, decreasing their hydrophobicity 3- to 5-fold compared to that of untreated controls and thereby significantly contributing to antibiofilm activity. Indeed, a positive correlation has been shown between cell-surface hydrophobicity and levels of biofilm formation^37^.

Our data demonstrate that the tested agents, and especially AEA, have a notably more pronounced effect on bacteria embedded in biofilm than on planktonic bacteria. This can be attributed to the specific non-bactericidal activity of the ECs, which modify the bacterial cell surface rather than destroying the bacterial cell. Indeed, both compounds are amphiphilic molecules, and AEA has been shown to interact with mammalian cell membranes via a non-specific receptor-independent mechanism^51^. It has been proposed that ECs can modify the lipid bilayer’s fluidity^52,53^ and elastic properties^51^. Since ECs non-specifically modify the eukaryotic membrane lipid bilayer, we hypothesized that these amphiphilic compounds would act similarly on the prokaryotic cell membrane lipid bilayer. It has been reported that small amphiphilic molecules can disturb established biofilms and affect bacteria by disrupting membrane integrity^54^. We found that AEA can lower the MP, causing membrane depolarization, albeit with much less impact than the proton ionophore CCCP, a depolarizing agent that destroys MP by eliminating the proton gradient. Intriguingly, in contrast to AEA, AraS caused hyperpolarization of the staphylococcal membrane by elevating MP. In addition to depolarization, hyperpolarization has also been documented to affect bacterial viability^55,56^. It seems that AraS at sub-MICs caused short-duration hyperpolarization of the MRSA CI strain membrane, which did not affect bacterial cell viability. Similarly, the antimicrobial peptide Bac8c at sub-killing concentrations has been shown to non-lethally destabilize the cytoplasmic membrane, resulting in at least transient hyperpolarization of the cytoplasmic membrane^56^.

A non-disruptive effect of the agents on MP indicates that they do not impair cell integrity, which clearly corresponds to their antibiofilm activity at sub-MICs. Moreover, we suggest that mechanism of anti-MRSA action of the tested compounds is attributed to modification of bacterial MP and subsequently alteration of biofilm-associated properties of MRSA, such as hydrophobicity and cell aggregation.

Taken together, our results demonstrate that the tested compounds (AEA in particular) are able to impair pathogenicity of MRSA by inhibiting biofilm formation, reducing metabolic activity of mature biofilm and modifying bacterial cell-surface characteristics without killing the bacteria.

Today, biofilm-associated staphylococcal infections are widespread. Beyond offering an environmental niche, biofilms also play an important role in the progression of chronic diseases. Recently utilized antibacterial agents aimed at treating bacterial infections are not capable of eradicating biofilms. Furthermore, the ability of bacterial pathogens to adapt to and overcome the challenges of antibiotics in their environment has been dramatically enhanced.

Therefore, we propose that ECs and EC-like compounds may serve as a natural line of defence against MRSA or other antibiotic-resistant bacteria. Due to their antibiofilm action, these agents could be a promising alternative to antibiotic therapeutics against biofilm-associated MRSA infections.

## Materials and Methods

### The tested compounds

**Figure 5:**
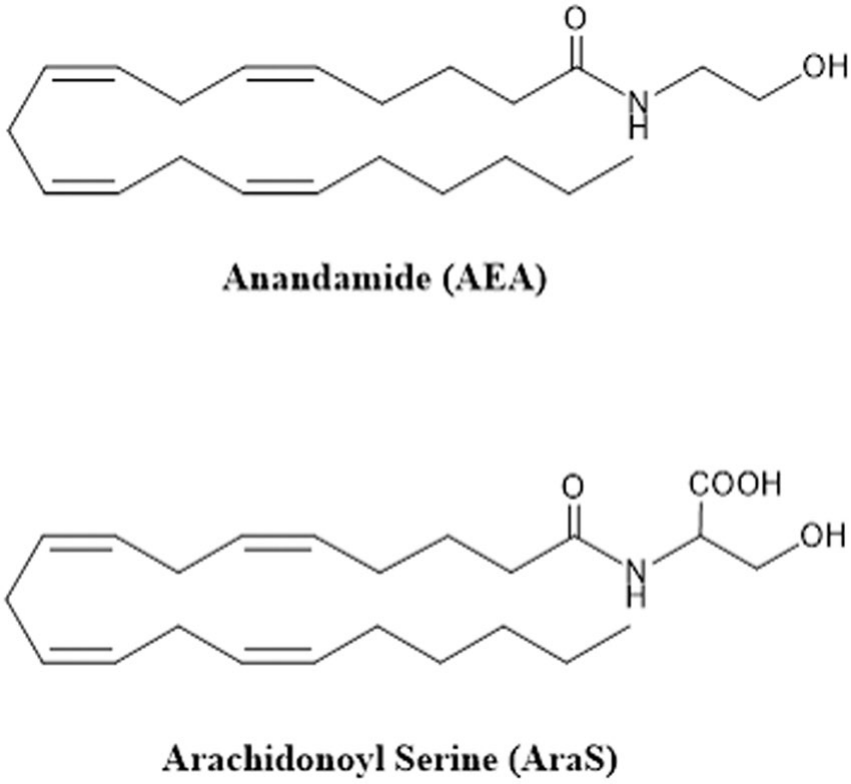
Structure of the compounds.

AEA was synthesized following the procedure described by Devane *et al*.^26^ and AraS was prepared following the procedure described by Milman *et al*.^27^ (Fig. 5).

### Preparation of bacterial inoculum

The bacteria used in this study were methicillin-resistant *Staphylococcus aureus* (MRSA) strains ATCC 33592, ATCC 43300, and a clinical isolate (CI). All bacterial strains were cultured from frozen stock in tryptic soy broth (TSB; Neogen, Lansing, MI, USA) and incubated at 37 °C for 24 h.

### Determination of minimal inhibitory concentration (MIC)

The MIC values of AEA and AraS against MRSA were determined using the twofold serial microdilution method based on the CLSI protocol^57^. The tested compounds were added to a 96-well plate containing sterile TSB medium. The range of final concentrations of all tested compounds was 4 µg/ml–256 µg/ml. Bacterial inoculum in the medium without the tested compound served as a positive control, whereas the tested compounds in medium without bacteria served as negative controls. The 96-well plate was then incubated at 37 °C for 24 h. The MIC was determined as the lowest concentration of the tested compound showing no turbidity after 24 h, where turbidity was interpreted as visible bacterial growth. The antibiotic gentamycin served as a control. The assay was performed in triplicate.

### Determination of minimal biofilm inhibitory concentration (MBIC)

The assay was performed as for the MIC evaluation except that the conditions were changed to a biofilm-formation-inducing environment by the addition of 1% glucose to the TSB medium. The antibiotic gentamycin served as a control. After incubation of bacteria with the tested compounds for 24 h, spent media and free-floating bacteria were removed by aspiration and the wells were carefully rinsed twice with phosphate-buffered saline (PBS, pH 7.4). Biofilm formation was quantified by crystal violet staining^46,58^. Briefly, 0.02% crystal violet was added to the wells and left for 45 min, and then the wells were washed twice with DDW to remove unbound dye. After adding 200 μl of 30% acetic acid to each well, the plate was shaken for 10 min to release the dye and the biofilm was quantified by measuring the absorbance at 595 nm using a Genios plate-reading spectrophotometer (Tecan, Salzburg, Austria). MBIC was determined as the lowest concentration of the tested compounds showing 90% biofilm inhibition compared to the untreated control. The assay was performed in triplicate.

### Effect of AEA and AraS on pre-formed biofilms

MRSA biofilms were allowed to mature in TSB + 1% glucose for 24 h at 37 °C in a 6-well plate. The biofilms were washed twice with PBS. AEA and AraS at two previously determined sub-MICs were then applied in TSB + 1% glucose to the mature biofilms. The plates were further incubated for 24 h at 37 °C. The amounts of MRSA biofilm were determined quantitatively using a standard 3-(4,5-dimethyl-2-thiazolyl)-2,5-diphenyl-2H-tetrazolium bromide (MTT) reduction assay as described previously^59–61^. Briefly, biofilm was overlaid with 100 mM MTT and incubated for 2 h at 37 °C. Under these conditions, the lightly yellowish MTT was reduced to a blue tetrazolium salt accumulating within the metabolically active biofilms. The stain was then dissolved in DMSO and the absorbance was measured at 570 nm. The accumulation of tetrazolium salt via the reduction of MTT is proportional to the number of metabolically active cells growing in the biofilm. The assay was performed in triplicate.

### Effect of AEA and AraS on MRSA spreading ability

The assay was performed as described previously^62^ in a 3-cm petri dish, on TSB containing 0.3% agar-agar powder and sub-MICs of the tested compounds. Dishes without tested compounds served as a control. Overnight cultures of the MRSA strains (OD_600_ = 0.6) were point-inoculated at the centre of the agar medium and incubated for 24 h at 37 °C. Spreading ability was determined by measuring the diameters of the spread and then comparing with the control. The assay was performed in triplicate.

### Effect of AEA and AraS on cell-surface hydrophobicity

Microbial surface hydrophobicity was assessed based on microbial adhesion to hydrocarbon using a previously described method^50,63^, in hexadecane as the organic solvent. Briefly, MRSA at a concentration of 10^7^ cell/ml was incubated for 30 min at 37° with or without the compounds at sub-MICs. Bacterial cells were then washed with PBS, suspended in the same buffer, and their OD_660_ determined. The cells were mixed with hexadecane (2.5:1), shaken for 2 min, and the tube was left for 20 min at room temperature for phase separation. The turbidity of the aqueous phase was read at 660 nm. The hydrophobicity index (HI) was calculated in percentage as HI = (OD_control_ − OD_test_) × 100/OD_control_, where OD_control_ = optical density at 660 nm before hexadecane treatment and OD_test_ = optical density at 660 nm after hexadecane treatment. The assay was performed in triplicate.

### Cell aggregation assay

Cell aggregation was analyzed as previously reported^64^ with some modifications. Briefly, MRSA cells (CI strain) were inoculated into 2 ml of TSB medium in 14-ml test tubes with sub-MICs of the tested compounds for 24 h with shaking at 250 rpm. Untreated sample served as a control. Cell cultures (1 ml) were then collected by centrifugation at 16,600 *g* for 2 min and cells were washed three times with PBS. Washed cells were resuspended in 3 ml PBS to OD_595_ = 1.5 (OD_initial_) in clean glass tubes and allowed to stand for 24 h at room temperature. Next, supernatants were gently aspirated and aggregated pellets were resuspended in 3 ml of PBS. Turbidity of aggregates was measured at OD_595_ (OD_final_) using the Genios plate-reading spectrophotometer. The percentage of aggregation was determined as: OD_final_/OD_initial_ × 100%. The relative aggregation of the treated samples was presented as percentage of that of the untreated control (100%). The assay was performed in triplicate.

### Effect of AEA and AraS on membrane potential (MP)

The ability of the tested compounds to affect the MP of the MRSA CI strain using the cationic dye 3,3′-diethyloxacarbocyanine iodide (DiOC_2_(3); Molecular Probes, Eugene, OR, USA) in a microtiter well-based assay was determined as described previously^65^ with some modifications. DiOC_2_(3) exhibits a shift in fluorescence from 500–575 nm (green) to >600 nm (red) in bacterial cells with an intact membrane. This red shift disappears when the bacterial membrane is damaged. Briefly, an overnight culture of MRSA was centrifuged, washed with PBS three times and further resuspended in PBS to a final concentration of OD_595_ = 3. Bacterial cells were incubated with DiOC_2_(3) at room temperature in the dark for 5 min. A 10-μl aliquot of this mixture was added to 40 μl reaction solution containing the tested compounds at sub-MICs or PBS (untreated control) and read immediately in an Infinite M200 Pro plate reader (Tecan). Excitation was read at 450 nm and emission at 690 nm. The difference in fluorescence between the tested compounds in treated and untreated control cells was detected at 690 nm and calculated as percentage of the untreated control (100%). The assay was performed in triplicate.

### Statistical analysis

Means of three independent experiments were calculated. The statistical analysis was performed using Student’s t-test with a significance level of *P* < 0.05 as compared to controls.
